# Meet the Editors‐in‐Chief

**DOI:** 10.1002/ansa.20190004

**Published:** 2020-03-24

**Authors:** Dietrich Volmer, Paul Trevorrow

**Affiliations:** ^1^ Humboldt‐Universität zu, Berlin; ^2^ Executive Journals Editor Wiley Chichester UK


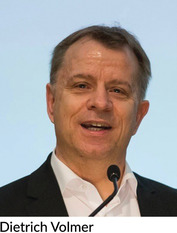


Dietrich Volmer is Full Professor and Chair in Bioanalytical Chemistry at Humboldt‐Universität zu Berlin. He graduated with a PhD in analytical chemistry at the University of Hannover in 1994.

After postdoctoral research as ORISE Fellow at the National Center for Toxicological Research in Jefferson, AR/USA, he joined the National Research Council's Institute for Marine Biosciences in Halifax, Nova Scotia in Canada in 1996 as a research associate.

He then spent a few years as Head of the Analytical Research Facility at Merck in Darmstadt, Germany, and subsequently returned to Canada in 2001, as Senior Research Officer and Group Leader Biological Mass Spectrometry at the Institute for Marine Biosciences in Halifax.

In 2007, Dietrich Volmer accepted a position as Head of the Department Bioanalytical Sciences of the Medical Research Council's Human Nutrition Research Institute in Cambridge, UK.

He became a full professor and Chair in Analytical Chemistry, and Director of the Institute of Bioanalytical Chemistry, at Saarland University in Saarbrücken, Germany in 2010, until he moved to his current position in Berlin in 2018. Dietrich Volmer has been Editor of Rapid Communications in Mass Spectrometry since 2004. He is a Fellow of the Royal Society of Chemistry and currently serves as Vice President of the German Society for Mass Spectrometry.

**Would you briefly explain what your research group is studying?**

My group focuses on several areas of bioanalytical chemistry and mass spectrometry, usually for small molecules, including metabolomics, quantification techniques, and instrument development. An area of particular current interest is the metabolism of vitamin D and the role of vitamin D in health and disease.

**Why did you choose a career in bioanalytical chemistry and mass spectrometry?**

My first encounter with analytical chemistry was during my PhD in the early 1990s, when I developed ultra‐trace analytical methodologies for unusual pesticides. We studied unusual pesticide compounds developed in the former East Germany. No monitoring methods existed for these compounds to measure their levels in the environment so we developed novel sensitive LC‐MS techniques. These were quite unusual back then. This was very exciting. My LC‐MS skills in the 1990s were in high demand and so it seemed logical to stay in that field.

**Of all your research projects, which one was your favorite and why?**

I am still a big fan of our efforts in trying to make MALDI mass spectrometry a quantitative technique for small molecules from biological samples, similar to LC‐MS. With all the benefits MALDI brings to the table; that is, simplicity and fast speeds. I do not think we really succeeded in convincing people back in the early 2000s but we are currently giving it another go by using our previous experience to improve the quantitative aspects of mass spectrometry imaging.

**What is your vision as editor on Analytical Science Advances?**

As Editor‐in‐Chief of Analytical Science Advances, my goal is to create a modern journal of high international standard and reputation that not only publishes top‐notch research in topical areas, but also follows current trends in open data and open access. I believe this is the perfect time for launching a journal such as this one instead of converting a previously established journal structure. As many authors today submit their analytical papers to more general science journals, I believe the time is right to win over these authors by providing a modern platform for open analytical science.

**What do you think is the key to success in a scientific career?**

I believe it is mainly persistence and really, *really* hard work. Intelligence is also required, but in my experience most successful people in academic or other research fields are pretty smart. Without the hard work, scientists will fail at some point, no doubt!

**Who were the most influential people in your career?**

The three people, who as advisors and mentors, always agreed with what I was interested in and let me get on with it! Few or no questions asked. These people were Karsten Levsen (University of Bonn), Jon Wilkes (National Center for Toxicological Research, Jefferson, AR), and Robert Boyd (National Research Council, Halifax, NS). I am very grateful to them!

**As a mentor and advisor, what do you advise your students in general?**

I tell every future student before they start their work with me that the most important aspect to being successful in science is to take ownership of the project that I give to them. This is important to create independent thinkers and project managers. The supervisor's main role is to give advice when needed (and to supply the technical and financial means to finish the research).

**What do you consider to be the more exciting topics in analytical chemistry?**

I am obviously biased in my answer, as I absolutely love everything that has to do with mass spectrometry. My areas of scientific excitement fluctuate with high frequency though, so any answer just represents a momentary snapshot. Currently, I favor imaging methods using various analytical techniques. In general, I believe that analytical technologies and methods will continue to strongly advance biology and medicine. At the same time, I expect environmental analytical chemistry to make a strong comeback, as climate change and a sustainable society will refocus priorities in the future.

**What are your views on the future of your field?**

My field of science – mass spectrometry – does not ever seem to run out of ideas. The upcoming challenges in the biological sciences will require major technical innovation in mass spectrometry and these innovations will be performed by chemists and physicists no doubt.

**What are your favorite past times outside of science?**

Since I have given a similar interview not too long ago, nothing has changed really, and I can only repeat that I have strong interest in the art of music reproduction. When enjoying this, I follow two completely opposing trends with equal obsession, analog vinyl and digital. In fact, sometimes the raw digital and analog data processing I perform with the music signals seems to resemble FTMS‐type signal processing routines… Obviously, in reality I have virtually zero time for this, as I usually enjoy spending time with my wife and young son.

**What would you do if you had 1‐year paid leave?**

Sitting in a Greek taverna all day, drinking coffee, and pretending that I work on the mass spectrometry textbook that I am planning to write for at least 10 years.

**What non‐scientist inspires you the most?**

As previously mentioned in a separate interview, none really. I really like Jerry Seinfeld though.

